# Sampling and Complementarity Effects of Plant Diversity on Resource Use Increases the Invasion Resistance of Communities

**DOI:** 10.1371/journal.pone.0141559

**Published:** 2015-11-10

**Authors:** Dan H. Zhu, Ping Wang, Wei Z. Zhang, Yue Yuan, Bin Li, Jiang Wang

**Affiliations:** Department Life Science, Taizhou University, Taizhou, Zhejiang, China; Shandong University, CHINA

## Abstract

**Background:**

Although plant diversity is postulated to resist invasion, studies have not provided consistent results, most of which were ascribed to the influences of other covariate environmental factors.

**Methodology/Principal Findings:**

To explore the mechanisms by which plant diversity influences community invasibility, an experiment was conducted involving grassland sites varying in their species richness (one, two, four, eight, and sixteen species). Light interception efficiency and soil resources (total N, total P, and water content) were measured. The number of species, biomass, and the number of seedlings of the invading species decreased significantly with species richness. The presence of *Patrinia scabiosaefolia* Fisch. ex Trev. and *Mosla dianthera* (Buch.-Ham. ex Roxburgh) Maxim. significantly increased the resistance of the communities to invasion. A structural equation model showed that the richness of planted species had no direct and significant effect on invasion. Light interception efficiency had a negative effect on the invasion whereas soil water content had a positive effect. In monocultures, *Antenoron filiforme* (Thunb.) Rob. et Vaut. showed the highest light interception efficiency and *P*. *scabiosaefolia* recorded the lowest soil water content. With increased planted-species richness, a greater percentage of pots showed light use efficiency higher than that of *A*. *filiforme* and a lower soil water content than that in *P*. *scabiosaefolia*.

**Conclusions/Significance:**

The results of this study suggest that plant diversity confers resistance to invasion, which is mainly ascribed to the sampling effect of particular species and the complementarity effect among species on resources use.

## Introduction

The pervasive impact of invasive species has motivated a considerable amount of research to question how the characteristics of invaded communities, such as native species diversity, affect the establishment of invasive species [1–5]. Elton [1] hypothesized that invasibility is determined by overall resource availability, which negatively relates to community diversity. Consistent with the hypothesis of Elton [1], most small-scale grassland experiments have shown that a more diverse plant community has a higher resistance to invasion by non-native plants [5–9]. However, other small-scale experiments have found that more diverse communities were not more resistant [10–15]. Consequently, the potential mechanisms by which plant communities resist invasion by non-native plants still need to be experimentally examined.

Resource availability and fluctuation are important factors in determining the invasion resistance of plant communities [16–17]. Tilman points out that species that utilize resources the least will gain advantage in competition [18]. Communities with higher species diversity could use a greater variety of resources, which results in greater use of the limiting resources, and hence are able to compete better with invaders [19]. Communities with higher plant diversity also have a higher probability of including a highly competitive species (which most likely has a strong demand for resources) and thus have a greater ability to resist invasion [20]. Moreover, some species have been proved to change resource conditions in a way that affects invasion by non-native plants [21–24]. Although the roles of species richness, species identity, and their effects on resource availability are central to understanding the invasion resistance of plant communities, these factors have rarely been investigated together.

In natural communities, changes in species diversity are usually accompanied by changes in environmental factors, which will, in turn, influence the effects of species diversity on invasion. Artificial microcosms have constant environments among communities and are ideal ecosystems for studying the mechanisms by which species diversity affects invasion. Moreover, early successional communities are ideal model systems for studying community invasibility because they are characterized by continuous species invasions, frequently and heavily invaded by exotic species, and typically invaded rapidly [25]. However, in most grassland microcosm experiments, non-native plants are introduced only once, by artificial seeding, which does not reflect the continuous exposure of a natural community to the pressure exerted by seeds of non-native plants. Moreover, invasions by native and non-native species do not fundamentally differ, and the ecological principles (e.g., niche limitation and competition for resources) underlying invasions by native and non-native species should be similar [5, 20, 25–26]. In this study, we manipulated grassland microcosms to ensure varying levels of species richness (one, two, four, eight, and sixteen species) and explored the resistance of communities to natural invasion by native and non-native plants. Based on the measurement of soil resource availability, we tested whether species diversity significantly increases the invasion resistance of communities, and, if it did, we examined the mechanism by which species diversity affects invasion resistance.

## Materials and Methods

### Ethics Statement

The experiments, conducted within the campus of Taizhou University, were pot based and therefore required no specific permission. The species defined as exotic in the present study were either natives or common exotic species that had already established local populations in the mountain area around Taizhou city, where Taizhou University is located. Consequently, none of the plant species used in this study is either endangered or protected.

### Experimental Materials

Sixteen common native species in the mountain area of Taizhou city in Zhejiang province of China were selected for the experiment (Table 1), and their seeds were collected in November 2012. Plastic containers (72 cm × 64 cm × 42 cm) were filled with yellow soil collected from the mountains (total N = 763.2 ± 103.69 mg kg^−1^) and the top 10 cm was filled with a mix containing equal proportions of the yellow soil and nutrient soil (mix ratio 1:1, total N = 4612.37 ± 456.23 mg kg^−1^). The plastic containers were used as experimental pots.

**Table 1:** 

Serial number	Names of planted species	Life form
**1**	*Antenoron filiforme* (Thunb.) Rob. et Vaut.	Perennial
**2**	*Achyranthes aspera* L.	Perennial
**3**	*Solanum nigrum* L.	Annual
**4**	*Penthorum chinense* Pursh	Perennial
**5**	*Sesbania cannabina* (Retz.) Poir.	Annual
**6**	*Patrinia scabiosaefolia* Fisch. ex Trev.	Perennial
**7**	*Eclipta prostrata* (L.) L.	Annual
**8**	*Polygonum chinense* L.	Perennial
**9**	*Bidens pilosa* L.	Annual
**10**	*Perilla frutescens* (L.) Britt. var. acuta (Thunb.)Kudo	Annual
**11**	*Mosla dianthera* (Buch.-Ham. ex Roxburgh) Maxim.	Annual
**12**	*Rostellularia procumbens* (L.) Nees	Annual
**13**	*Polygonum lapathifolium* L. var. salicifolium Sibth.	Annual
**14**	*Lolium perenne* L.	Perennial
**15**	*Cichorium intybus* L.	Perennial
**16**	*Medicago sativa* L.	Perennial

The names of planted species

### Experimental Design

In March 2013, 204 grassland pots with different levels of species richness (one, two, four, eight, and sixteen) were established. All the sixteen species used in the experiment were grown in monocultures without replication. Ten mixtures, each with a different species composition, were established to represent species richness levels of two, four, and eight species, and one mixture, replicated five times, represented a species-richness level of sixteen species (S1 Table). The species assigned to each mixture were chosen by a separate random draw of the appropriate number of species from the species pool. We introduced 800 seeds into each pot, and the number of seeds (and later the number of seedlings) for each species was determined by dividing 800 by the number of species per pot. The plant density in each pot was maintained at 32 seedlings. One month after germination, excess seedlings were removed, retaining the more vigorous seedlings. Seedlings of the same species were not adjacent, and 32 seedlings were evenly distributed in the pot. The complete experiment design was replicated four times, each replication constituting a block. Seedlings of non-target species were removed to maintain the original composition of the community. As frequent removal of seedlings was a disturbance, they were removed before they were large enough to influence the community. Besides natural rainfall, each block received the same amount of water through sprinkler irrigation every 4 days during dry spells.

### Invasion Experiment

One year later, in March 2014, the non-target seedlings were removed for the last time from each pot, and the invaders were recorded then on as the seedlings emerged from the soil seed bank or from seeds outside the experimental pots. In order to avoid the influence of interactive invasion between pots, any seedlings of the sixteen experimental species that had not been included originally in each pot were removed every week during the invasion experiment. In September 2014, the number of seedlings of each non-target native species (native species that were not among the sixteen planted species) and exotic species was counted. The total biomass of all living seedlings was sorted according to each non-target species, dried to a constant mass at 80°C for 48 h, and weighed.

### Light and Soil Water

Photosynthetically active radiation (PAR) was measured using a PAR ceptometer (GLZ-C, Zhejiang Top Instrument Co. Ltd., Nibo, China). To avoid edge effects, three points were randomly selected in the central area (0.4 m × 0.4 m) of each pot and the electronic fisheye sensor was covered with a black cloth. The top 1 cm was left uncovered. We took measurements during 11:30–13:00 when the sun was more or less overhead and the pots received rays that were near vertical. On cloud-free days in April, June, and August 2014, PAR above the community canopy and that at ground level were measured in triplicate at each of the three points. The light interception efficiency (*LIE*) of the pot was estimated as follows:

$$LIE=(PAR_{above}-PAR_{ground})/PAR_{ground}$$

The average *LIE* of the three points measured in triplicate was used as the *LIE* of each pot.

Before surveying the non-target native and exotic species, a water treatment experiment was conducted. The water content of each pot was determined 3 days after they had been irrigated. The water content (%, m^3^/m^3^) at each of the three points was measured at 15:00–16:00 using a ProCheck (Decagon, Pullman, Washington, USA) analyzer. The average water content of the three points measured in triplicate was used as the water content for each pot.

### Total N and Total P

In March 2014, five soil cores (0–20 cm) were randomly collected and pooled to make one sample. The soil sample was air-dried, homogenized, sieved (<2 mm) to remove plant roots and small stones, and used for determining the total N and total P in the soil. For these determinations, 1.0 g of the K_2_SO_4_ catalyst mixture and 5 mL of concentrated H_2_SO_4_ were added to 0.5 g of air-dried natural or mine tailing soil in 100-mL digestion tubes. After heating the mix to a milky white color, 20 mL of distilled water was added to the digestion tube. Total N was determined using the Berthelot reaction method and total P using the molybdenum blue method [27]. The total N and total P were measured in three replications per pot.

### Statistical Analyses

We analyzed two types of invader species: non-target native species and exotic species. Non-target native species are species that were known to grow in the region but were not among the sixteen native species chosen for the experiment whereas exotic species are species that never grew in the region before and were invaders from other places. Total invaders in each pot comprised all the non-target native species and the exotic species. The number of species, biomass, and the number of seedlings of total and exotic invaders were analyzed using a general linear model (GLM) with block as a random factor and species richness as a fixed factor. The relationship between these variables and planted diversity was determined by including planted-species richness as a factor in a regression analysis combined with curve estimation. The effects of particular species were determined in two steps. First, differences in invasibility between monocultures were determined using a GLM with block as a random factor and species identity as a fixed factor. Second, we selected species that differed markedly from other species according to those tests and included the presence of these species as covariates in the extended GLM model. The interaction term between richness and the presence of particular species were also included in the extended GLM model. This procedure was repeated for six variables: the number of species, biomass, and the number of seedlings of both total and exotic invaders.

**Fig 1:**
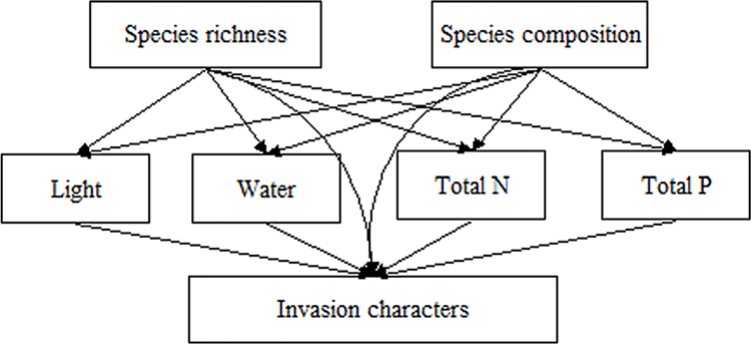
A priori structural equation model representing hypothesized relationships among the species richness, species composition, resources (light, soil water content, total N and total P) and invasion characters (species number, biomass and seedling number of total and exotic invaders).

Structural equation modeling (SEM) is a multivariate method that allows explicit testing of direct and indirect dependencies and is therefore well suited for assessing the direct and indirect effects of species richness, species composition, and resources on invasion (Fig 1). Prior to the SEM, all variables were normalized by subtracting the mean and dividing by the standard deviation. We assessed whether the values derived from SEM fitted the data by a series of goodness-of-fit tests, which compared the observed covariance matrix with that derived from the model [28]. First, we performed a *χ* ^2^ test to evaluate the goodness-of-fit of our model. We also performed goodness-of-fit tests, such as the goodness-of-fit index (GFI) and the Bentler–Bonett normed-fit index (NFI) [28–29]; both range between 0 and 1, with values greater than 0.90 indicating a good fit.

The light interception efficiency of mixtures was compared to that of the monoculture with the highest light interception efficiency, and the soil water content of mixtures was compared to that of the monoculture with the lowest soil water content. The analysis addressed the following question: Does the percentage of pots with light interception efficiency greater than the highest light interception efficiency or that of pots with soil water content lower than the lowest value increase with the species richness? The relationships between the percentage of pots and species richness was analyzed using bivariate correlation analysis. All analyses were performed using SPSS ver. 20.0 for Windows and the SEM analysis was conducted using AMOS ver. 20.0, which is part of SPSS 20.0 (Amos Development Corp., Mount Pleasant, South Carolina, USA).

## Results

**Fig 2:**
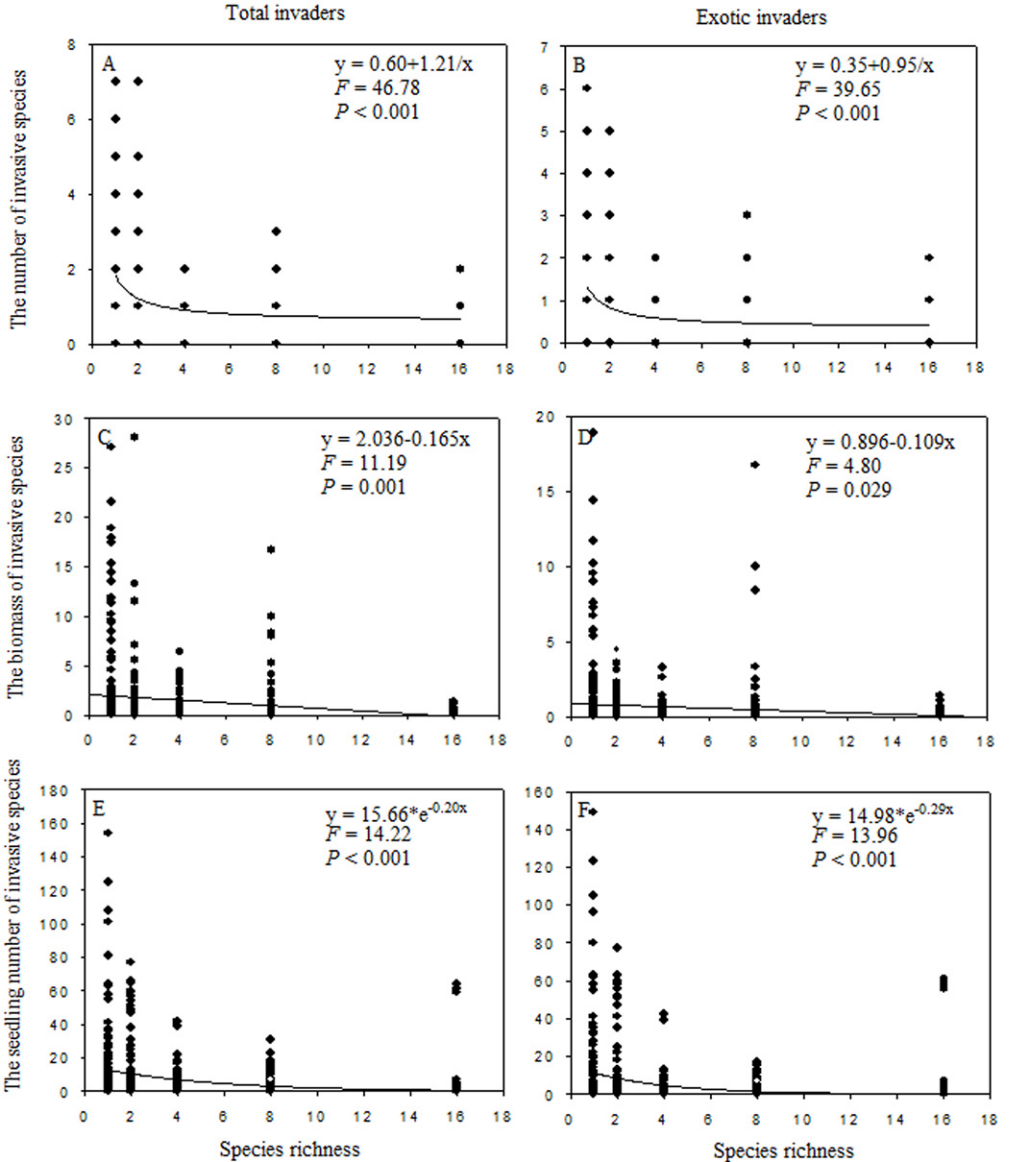
The dependence of species number (A, B), biomass (C, D) and seedling number (E, F) of total and exotic invaders on species richness.

The survey of invader species found six exotic species and 23 non-target native species (S2 Table). The species number, biomass, and seedlings number of both total and exotic invaders were strongly affected by the planted-species richness (Table 2). The biomass of the total and exotic invaders decreased linearly with planted-species richness (Fig 2A and 2B). The species number (Fig 2C and 2D) and the seedlings number (Fig 2E and 2F) of total and exotic invaders decreased non-linearly with the planted-species richness.

**Table 2:** 

	Species number of total invaders	Species number of exotic invaders	Biomass of total invaders	Biomass of exotic invaders	Seedling number of total invaders	Seedling number of exotic invaders
**Block**	1.973^ns,^ ^α^	2.629^ns^	0.896^ns^	1.501^ns^	2.519^ns^	2.502^ns^
**Species richness**	12.807***	10.708***	5.145***	4.52**	5.592***	5.508***

The effects of block and species richness on species number, biomass and seedling number of total and exotic invaders.

^α^ Significant terms are indicated by asterisks (**, *P* < 0.01; ***, *P* < 0.001) and unsignificant terms are indicated by “ns”.

**Fig 3:**
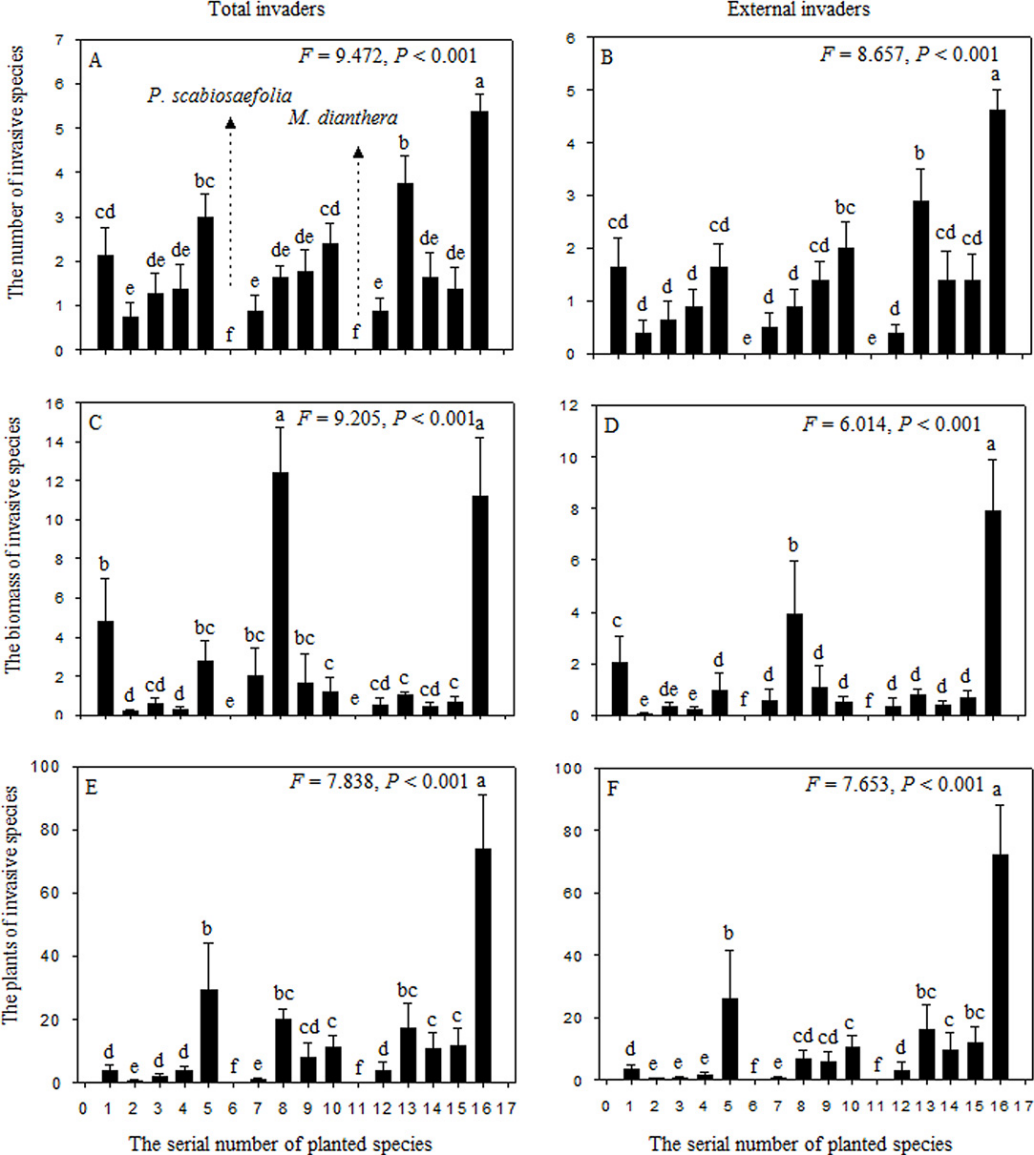
The species number, biomass and seedling number of total and exotic invaders in monocultures. Data show means ± SE. The serial number of planted species is seen in Table 1. Monocultures had not at least one same letter indicate significant difference.

Invasibility differed greatly between monocultures of planted species (Fig 3A–3F). The monocultures of *P*. *scabiosaefolia* and *M*. *dianthera* were not invaded by the exotic and non-target native species and these two species had the highest invasion resistance. The presence or absence of *P*. *scabiosaefolia* and *M*. *dianthera* was additionally included in the GLM model. The species number, biomass, and the seedlings number of total and exotic invaders were negatively affected by the presence of *P*. *scabiosaefolia* and *M*. *dianthera* (Table 3), but a significant negative effect of planted-species richness remained. The interaction between planted-species richness and the presence of *P*. *scabiosaefolia* and *M*. *dianthera* had no significant effect on the invasion by exotic and non-target native species.

**Table 3:** 

	Species number of total invaders	Species number of exotic invaders	Biomass of total invaders	Biomass of exotic invaders	Seedling number of total invaders	Seedling number of exotic invaders
**Block**	2.197^ns,^ ^α^	2.353^ns^	0.911^ns^	1.517^ns^	2.362^ns^	2.137^ns^
**Species richness (*SR*)**	9.143↓^β^ ^, ***^	8.103↓***	3.904↓**	5.067↓**	3.674↓*	4.221↓**
***P*.*scabiosaefolia***	15.867↓***	13.765↓***	4.299↓*	4.019↓*	6.628↓*	4.658↓*
***M*.*dianthera***	30.795↓***	23.104↓***	7.715↓**	5.839↓*	12.191↓**	8.303↓**
***SR*×*P*.*scabiosaefolia***	2.235^ns^	2.463^ns^	0.859^ns^	0.818^ns^	0.966^ns^	0.868^ns^
***SR*×*M*.*dianthera***	2.226^ns^	2.019^ns^	0.602^ns^	0.506^ns^	0.993^ns^	1.289^ns^

Results of General Linear Model (type III SS) on six separate variables (Species number, biomass and seedling number of total and exotic invaders). Block and species richness was included as a factor. For the presence of *P*. *scabiosaefolia* and *M*. *dianthera* dummy variables were created. Values could either be zero (absent) or one (present). Dummy variables were also included as covariates.

^α^ Significant terms are indicated by asterisks (*P < 0.05; **P < 0.01; ***P < 0.001) and unsignificant terms are indicated by “ns”.

^β^ ↓ indicates had positive effect.

**Fig 4:**
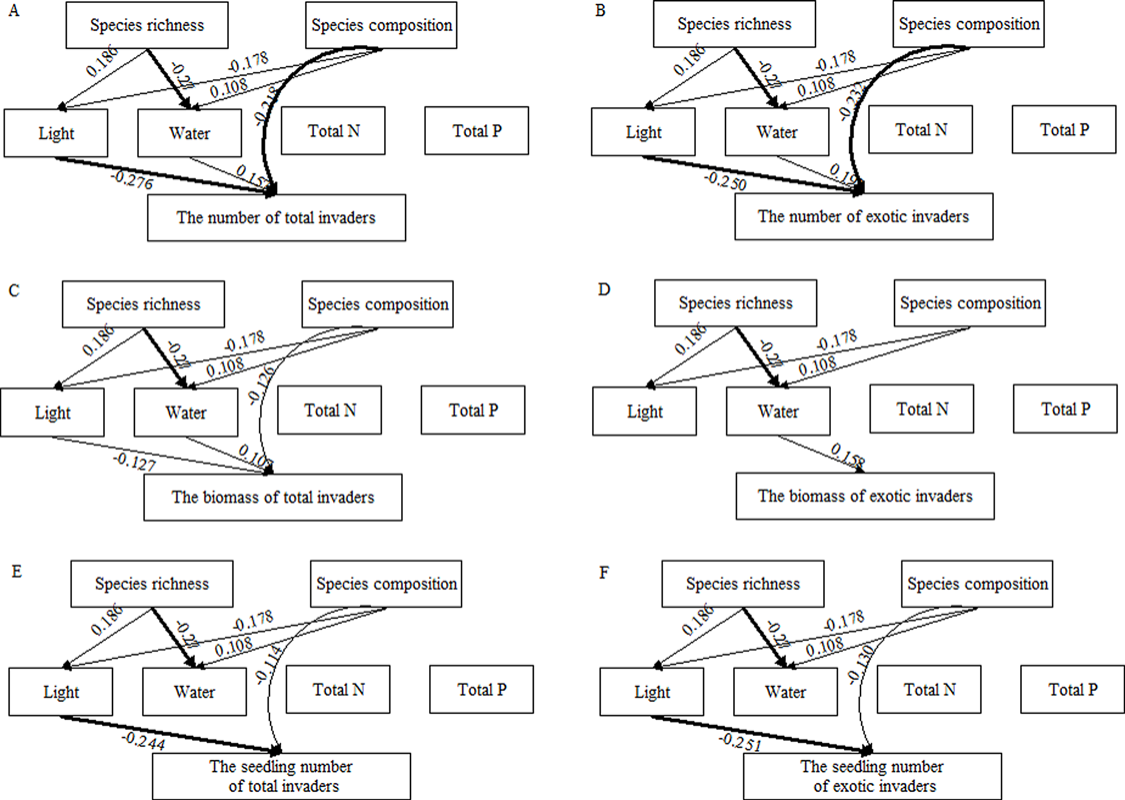
The final structural equation models for species number (A, B), biomass (C, D) and seedling number (E, F) of total and exotic invaders. Values on arrows represent standardized regression coefficients. Continuous arrows, in the structural part of the model, are significant relationships (*P* < 0.05); The arrows not significant are eliminated (*P* > 0.05). The size of the arrows is proportional to the strength of the path. The significant relationships of species composition only indicate significant effect on invasion, and the “-” did not indicate a negative effect.

For the SEM models, the *χ* ^2^ test was not significant at *P* > 0.05, and GFI and NFI values were higher than 0.90, indicating the goodness-of-fit to the data. In the SEM models, species richness had no effect on the characteristics of the invasion (Fig 4). Except for the biomass of exotic invaders, light interception efficiency negatively affected all the characteristics of invasion. Except for the plants of total and exotic invaders, soil water content positively affected the number and biomass of total and exotic invaders. Except for the biomass of exotic invaders, species composition significantly affected all the characteristics of invaders, probably because of the effect of particular species on light interception efficiency and soil water content. The presence of *P*. *scabiosaefolia* and *M*. *dianthera* significantly increased the light interception efficiency (*P*. *scabiosaefolia*: *t* = 2.898, *n* = 202, *P =* 0.004; *M*. *dianthera*: *t* = 2.142, *n* = 202, *P =* 0.042) but significantly decreased the soil water content (*P*. *scabiosaefolia*: *t* = −3.870, *n* = 202, *P* < 0.001; *M*. *dianthera*: *t* = −5.185, *n* = 202, *P* < 0.001).

**Fig 5:**
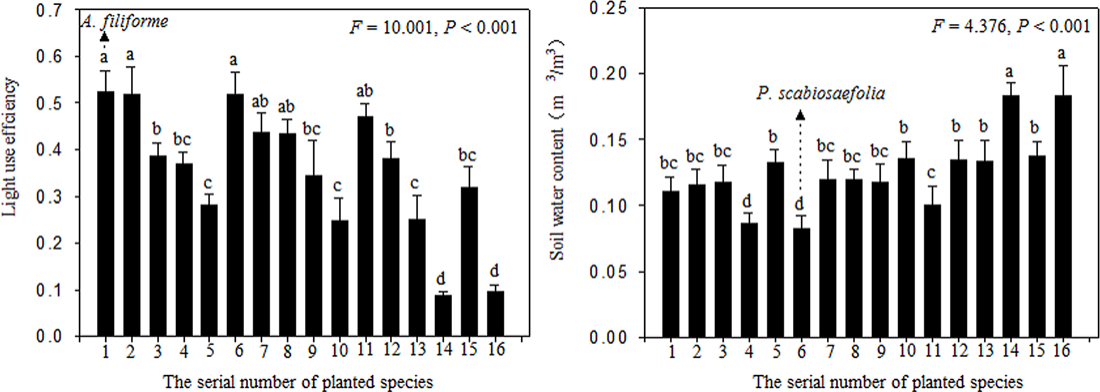
Light interception efficiency and soil water content in monocultures. Data show means ± SE. The serial number of planted species is seen in Table 1. Monocultures had not at least one same letter indicate significant difference.

**Fig 6:**
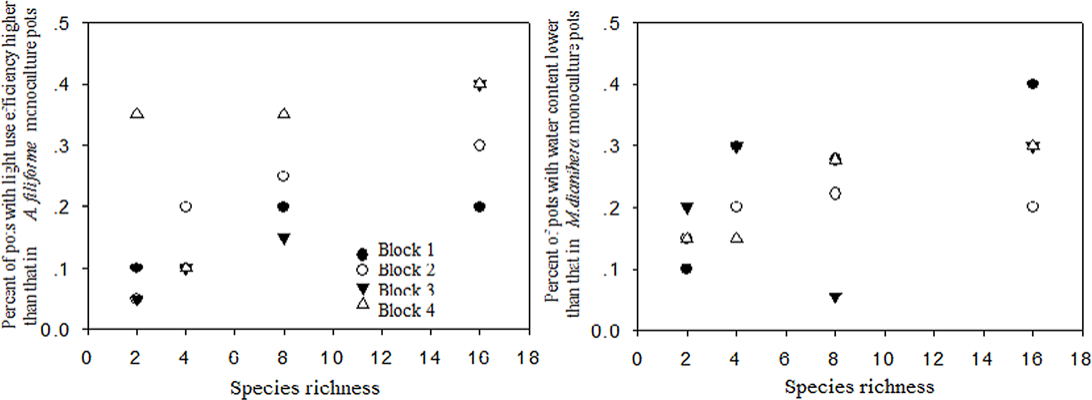
Percent of pots with light interception efficiency higher than that of *A*. *filiforme* monocultures and percent of pots with soil water content lower than that of *P*. *scabiosaefolia* monocultures.

The negative effect of *P*. *scabiosaefolia* and *M*. *dianthera* on invasion suggests a sampling effect, and the complementary effect was also investigated based on resource use. The species with the highest light interception efficiency as a monoculture was *A*. *filiforme*. The species with the lowest soil water content as a monoculture was *P*. *scabiosaefolia* (Fig 5). We used the light interception efficiency values from the monocultures of *A*. *filiforme* and the soil water content values from the monocultures of *P*. *scabiosaefolia* as cut-off values. We calculated for each species-diversity treatment the percentage of pots with light interception efficiency above the cut-off value and the percentage of pots with soil water content below the cut-off (Fig 6). We found that as planted-species richness increased, there was a highly significant increase in the percentage of pots with light interception efficiency higher than that *A*. *filiforme* monoculture (Fig 6, *r* = 0.664, *n* = 16, *P* = 0.005) and also the percentage of pots with soil water content lower than that in *P*. *scabiosaefolia* monoculture (*r* = 0.553, *n* = 16, *P* = 0.033).

## Discussion

Our results are consistent with earlier invasion experiments, which have shown negative effects of species richness on invasion [5, 9, 20, 30–33]. The extended GLM models showed that planted-species richness had significant negative effects on invasion, which indicates that after taking into account the presence of suppressive species (*P*. *scabiosaefolia* and *M*. *dianthera*), other mechanisms related to plant diversity still decreased the invasion. The negative effect of diversity is often explained by increased resource use, whereby complementarity of resource use between species results in lower levels of available resources at high diversity, thus inhibiting invasion [33–36]. In the analysis of SEM, species richness had no direct effect on invasion, whereas light interception efficiency affected the invasion negatively and soil water content affected the invasion positively. Moreover, the presence of *P*. *scabiosaefolia* and *M*. *dianthera* significantly increased light interception efficiency and decreased soil water content, which led to the significant effect of species composition on invasion. Therefore, our results suggest that in this study the ability of plant communities to efficiently consume light and soil water should be a key factor determining the invasion resistance of communities. Total N (seen in methods) and total P most likely increased because of the addition of nutrient soil in our study, and most probably the increase eliminated any significant effect of plant communities on total N and total P. Because the limiting resource should be the key factor affecting invasion [18], it was little affected by total N and total P.

Our extended GLM models revealed that plant identity is an important factor that influences the invasibility of communities [32, 37–39]. In this study, *P*. *scabiosaefolia* and *M*. *dianthera* had the highest invasion resistance among the sixteen monocultures. A decrease in invasion with an increase in planted-species richness might be a result of the increased frequency of *P*. *scabiosaefolia* or *M*. *dianthera* in pots from monoculture to the richness level represented by the sixteen species, which suggests a strong sampling effect that contributes to invasion resistance in diverse communities. Although *A*. *filiforme* had the highest light interception efficiency, the presence *A*. *filiforme* had no negative effect on invasion. *P*. *scabiosaefolia* and *M*. *dianthera* simultaneously had high light interception efficiency and low soil water content (Fig 5), which suggest that invasion resistance might be an overall result of the constraints on the availability of different resources.

In addition to the sampling effect, we detected a clear signature of the complementarity effect: the overuse of light and soil water. Such overuse occurs when species-diverse pots use resources more efficiently than the best species in a monoculture, indicating a complementarity effect of plant diversity on resource use [40]. Stachowicz et al. [41] and Fargione and Tilman [20] also found that the percentage of pots with invader biomass lower than the lowest invader biomass in monocultures increased with diversity. This demonstrates that increasing species richness will increase the invasion resistance of communities beyond what can be achieved even by the strongest competitor alone (e.g., *P*. *scabiosaefolia* and *M*. *dianthera* in this study). As the use of light and of soil water were the key factors affecting resistance to invaders in this study, its results indicate that interspecific complementarity in resource use in diverse pots contributes to greater inhibition of invaders.

Plant litter may be an important factor that influences invasion [42], and has been shown to constrain the germination of invader seeds [43–44]. In the present study, *L*. *perenne* and *M*. *sativa* shed their leaves in late May, whereas other planted species did not shed their leaves before the survey. We did not collect data on litter composition, but we observed that most of the *L*. *perenne* litter remained in the pots until the survey and that most of *M*. *sativa* litter disappeared within 1 month. Moreover, *L*. *perenne* monoculture has a high invasion resistance, while *M*. *sativa* has a low invasion resistance (Fig 2). The presence of *L*. *perenne* also significantly decreased the species number, biomass, and the seedlings number of all invaders, and the number of species of exotic invaders (data not shown). Consequently, litter accumulation may be also an important factor influencing invasion resistance.

The evidence presented in this study suggests that the sampling and complementarity effects are strong mechanisms of diversity that influence invasion resistance. Moreover, species composition is also an important factor influencing invasibility of communities. Consequently, the loss of plant diversity and key species may greatly decrease the ability of communities to resist invasion. Although the non-target native species were included in our study to illustrate the effect of plant diversity on invasion resistance, the establishment of non-target native species and the invasion of exotic species are to a large extent affected by the same mechanisms [20, 26]. Therefore, plant diversity may have similar effects on invasion by exotic species [45]. Although we examined the establishment of invader species only for 1 year, seedling establishment is a key life-stage for invaders [46–48]. Understanding the effect of such initial environmental ‘filters’ on invasion may aid in the control of natural or human-induced invasions.

## Supporting Information

S1 Table The species composition of experimental pots. (DOC)

S2 Table The name of non-target native and exotic species. (DOC)

## References

[1] Elton CS (1958) The ecology of invasions by animals and plants. Methuen, London, UK.

[2] Vitousek PM, Mooney HA, Lubchenco J, Melillo JM (1997) Human domination of Earth’s ecosystems. Science 277: 494–499.

[3] Levine JM, D’Antonio CM (1999) Elton revisited: a review of evidence linking diversity and invasibility. Oikos 87: 15–26.

[4] Tilman D (1999) The ecological consequences of changes in biodiversity: a search for general principles. Ecology 80: 1455–1474.

[5] Roscher C, Beßler H, Oelmann Y, Engels C, Wilcke W, Schulze E (2009) Resources, recruitment limitation and invader species identity determine pattern of spontaneous invasion in experimental grasslands. J Ecol 97: 32–47.

[6] Dimitrakopoulos PG, Galanidis A, Siamantziouras AD, Troumbis AY (2005) Short-term invasibility patterns in burnt and unburnt experimental Mediterranean grassland communities of varying diversities. Oecologia 143: 428–437. 15711823 10.1007/s00442-004-1808-8

[7] Maron J, Marler M (2007) Native plant diversity resists invasion at both low and high resource levels. Ecology 88: 2651–2661. 18027767 10.1890/06-1993.1

[8] Mwangi PN, Schmitz M, Scherber C, Roscher C, Schumacher CJ, Scherer-lorenzen M, et al (2007) Niche pre-emption increases with species richness in experimental plant communities. J Ecol 95: 65–78.

[9] Byun C, de Blois S, Brisson J (2013) Plant functional group identity and diversity determine biotic resistance to invasion by an exotic grass. J Ecol 101: 128–139.

[10] Lonsdale WM (1999) Global patterns of plant invasions and the concept of invasibility. Ecology 80: 1522–1536.

[11] Stohlgren TJ, Binkley D, Chong GW, Kalkhan MA, Schell LD, Bull KA, et al (1999) Exotic plant species invade hot spots of native plant diversity. Ecol Monogr 69: 25–46.

[12] Levine JM (2000) Species diversity and biological invasions: relating local process to community pattern. Science 288: 852–854. 10797006 10.1126/science.288.5467.852

[13] Knight KS, Oleksyn J, Jagodzinski AM, Reich PB, Kasprowicz M (2008) Overstorey tree species regulate colonization by native and exotic plants: a source of positive relationships between understorey diversity and invisibility. Divers Distrib 14: 666–675.

[14] Kellner JB, Hastings AA (2009) Reserve paradox: introduced heterogeneity may increase regional invisibility. Conserv Lett 2: 115–122.

[15] Souza L, Bunn WA, Simberloff D, Lawton RM, Sanders NJ (2011) Biotic and abiotic influences on native and exotic richness relationship across spatial scales: favourable environments for native species are highly invasible. Funct Ecol 25: 1106–1112.

[16] Davis MA, Grime GP, Thompson K (2000) Fluctuating resources in plant communities: a general theory of invasibility. J Ecol 88: 528–534.

[17] Tilman D (2004) Niche tradeoff s, neutrality, and community structure: a stochastic theory of resource competition, invasion, and community assembly. Proc Natl Acad Sci USA 101: 10854–10861. 15243158 10.1073/pnas.0403458101 PMC503710

[18] Tilman D (1999). The ecological consequences of changes in biodiversity: a search for general principles. Ecology 80: 1455–1474.

[19] Hooper DU, Chapin FS, Ewel JJ, Hector A, Inchausti P, Lavorel S, et al (2005) Effects of biodiversity on ecosystem functioning: a consensus of current knowledge. Ecol Monogr 75: 3–35.

[20] Fargione JE, Tilman D (2005) Diversity decreases invasion via both sampling and complementarity effects. Ecol Lett 8: 604–611.

[21] Smith MD, Wilcox JC, Kelly T, Knapp AK (2004) Dominance not richness determines invasibility of tallgrass prairie. Okios 103: 253–262.

[22] Emery SM, Gross KL (2006) Dominant species identity regulates invasibility of old-field plant communities. Okios, 115: 549–558.

[23] Emery SM (2007) Limiting similarity between invaders and dominant species in herbaceous plant communities? J Ecol 95: 1027–1035.

[24] Losure DA, Wilsey BJ, Moloney KA (2007) Evenness-invasibility relationships differ between two extinction scenarios in tallgrass prairie. Okios, 116: 87–98.

[25] Meiners SJ, Cadenasso ML, Steward T, Pickett A (2004) Beyond biodiversity: individualistic controls of invasion in a self-assembled community. Ecol Lett 7: 121–126.

[26] van Ruijven J, De Deyn GB, Berendse F (2003) Diversity reduces invasibility in experimental plant communities: the role of plant species. Ecol Lett 6: 910–918.

[27] Page AL, Miller RH, Keeney DR (1982) Methods of soil analysis chemical and microbiological properties. Madison, Wisconsin.

[28] Shipley B (2002) Cause and correlation in biology A user’s guide to path analysis, structural equations and causal inference. Cambridge University Press, Cambridge, UK.

[29] Iriondo JM, Albert MJ, Escudero A (2003) Structural equation modelling: an alternative for assessing causal relationships in threatened plant populations. Biol Conserv 113: 367–377.

[30] Hector A, Dobson K, Mins A, Bazeley-White E, Lawton JH (2001) Community diversity and invasion resistance: an experimental test in a grassland ecosystem and a review of comparable studies. Ecol Res 16: 819–831.

[31] Lyons KG, Schwartz MW (2001) Rare species loss alters ecosystem function–invasion resistance. Ecol Lett 4: 358–365.

[32] van Ruijven J, Berendse F (2003) Positive effects of plant species diversity on productivity in the absence of legumes. Ecol Lett 6: 170–175.

[33] Reinhart KO, Maestre FT, Callaway RM (2006) Facilitation and inhibition of seedlings of an invasive tree(*Acer platanoides*) by different tree species in a mountain ecosystem. Biol Inva 8: 231–240.

[34] Knops JMH, Tilman D, Haddad NM, Naeem S, Mitchell CE, Haarstad J (1999) Effects of plant species richness on invasion dynamics, disease outbreaks, insect abundances and diversity. Ecol Lett 2: 286–293. 10.1046/j.1461-0248.1999.00083.x 33810630

[35] Naeem S, Knops JMH, Tilman D, Howe KM, Kennedy T, Gale S (2000) Plant diversity increases resistance to invasion in the absence of co varying extrinsic factors. Oikos 91: 97–108.

[36] Mason TJ, French K, Russell K (2012) Are competitive effects of native species on an invader mediated by water availability? J Veg Sci, 23: 657–666.

[37] Crawley MJ, Brown SL, Heard MS, Edwards GR (1999) Invasion-resistance in experimental grassland communities: species richness or species identity? Ecol Lett 2: 140–148.

[38] Van der Putten WH, Mortimer SR, Hedlund K, Van Dijk C, Brown VK, Leps J (2000) Plant species diversity as a driver of early succession in abandoned fields: a multi-site approach. Oecologia 124: 91–99. 28308417 10.1007/s004420050028

[39] Wardle DA (2001) Experimental demonstration that plant diversity reduces invasibility–evidence of a biological mechanism or a consequence of sampling effect? Oikos 95: 161–170.

[40] Hector A, Bazeley-White E, Loreau M, Otway S, Schmid B (2002) Overyielding in grassland communities: testing the sampling effect hypothesis with replicated biodiversity experiments. Ecol Lett 5: 502–511.

[41] Stachowicz JJ, Fried H, Osman RW, Whitlatch RB (2002) Biodiversity, invasion resistance, and marine ecosystem function:reconciling pattern and process. Ecology 83: 2575–2590.

[42] Schuster MJ, Dukes JS (2014) Non-additive effects of invasive tree litter shift seasonal N release: a potential invasion feedback. Oikos 123: 1101–1111.

[43] Allison S, Vitousek P (2004) Rapid nutrient cycling in leaf litter from invasive plants in Hawai’i. Oecologia 141: 612–619. 15549401 10.1007/s00442-004-1679-z

[44] Liao CZ, Peng RH, Luo YQ, Zhou XH, Wu XW, Fang CM, et al (2008) Altered ecosystem carbon and nitrogen cycles by plant invasion: a meta-analysis. New Phytol 177: 706–714. 18042198 10.1111/j.1469-8137.2007.02290.x

[45] Kennedy TA, Naeem S, Howe KM (2002) Biodiversity as a barrier to ecological invasion. Nature 417: 636–638. 12050662 10.1038/nature00776

[46] Silvertown J, Franco M, Pisanty I (1993) Comparative plant demography relative importance of life-cycle components to the finite range of increase in woody and herbaceous perennials. J Ecol 81: 465–476.

[47] Shea K, Kelly D (1998) Estimating biocontrol agent impact with matrix models: Carduus nutans in New Zealand. Ecol Appl 8: 824–832.

[48] Parker IM (2000) Invasion dynamics of *Cytisus scoparius*: a matrix model approach. Ecol Appl 10: 726–743.

