# Correction: Sampling and Complementarity Effects of Plant Diversity on Resource Use Increases the Invasion Resistance of Communities

**DOI:** 10.1371/journal.pone.0150128

**Published:** 2016-02-19

**Authors:** Dan H. Zhu, Ping Wang, Wei Z. Zhang, Yue Yuan, Bin Li, Jiang Wang

The affiliation for the sixth author is incorrect. Jiang Wang is affiliated not only with #1, but also with Zhejiang Provincial Key Laboratory of Plant Evolutionary Ecology and Conservation, Taizhou University, Taizhou, China.
