# Supplementary material for: Sampling and Complementarity Effects of Plant Diversity on Resource Use Increases the Invasion Resistance of Communities
Source: PLoS One. 2015 Nov 10;10(11):e0141559. doi: 10.1371/journal.pone.0141559 (PMC4640883; doi:10.1371/journal.pone.0141559)
Supplement: S1 Table — (DOC) [file pone.0141559.s001.doc]

**S1Table.**

| **Species richness** | | | | |
| --- | --- | --- | --- | --- |
| **1** | **2** | **4** | **8** | **16** |
| ***Antenoron filiforme* (Thunb.) Rob. et Vaut. (AF)** | AA+PL | AF+PS+AA+MS | SC+CI+BP+PC+PS+MD+PF+LP | All 16 species |
| ***Achyranthes aspera* L. (AA)** | MD+PC | SN+LP+CI+PL | MS+PF+LP+SC+RP+SN+AA+MD |
| ***Solanum nigrum* L. (SN)** | AA+PF | AF+PL+CI+PC | PS+AF+LP+PL+SN+PF+BP+RP |
| ***Penthorum chinense* Pursh (PC)** | CI+SC | PC+RP+PF+EP | PC+RP+SN+PS+PC+LP+CI+BP |
| ***Sesbania cannabina* (Retz.) Poir. (SC)** | PF+BP | AF+MD+EP+SC | PC+PS+RP+PC+SC+AA+PF+AF |
| ***Patrinia scabiosaefolia* Fisch. ex Trev. (PS)** | SC+PL | RP+PC+MD+PF | RP+AF+EP+PL+AA+BP+PS+SN |
| ***Eclipta prostrata* (L.) L. (EP)** | PS+CI | BP+AA+MS+PL | SC+MD+CI+PF+PS+PC+AA+PC |
| ***Polygonum chinense* L. (PC)** | PS+PF | PF+PL+BP+LP | LP+SN+PS+PF+AF+MD+RP+BP |
| ***Bidens pilosa* L. (BP)** | MS+PL | CI+PS+PF+PC | PC+MS+AF+EP+LP+PS+PF+PC |
| ***Perilla frutescens* (L.) Britt. var. acuta (Thunb.)Kudo (PF)** | SN+PC | PF+SC+CI+AF | RP+AF+EP+PL+SN+PF+BP+RP |
| ***Mosla dianthera* (Buch.-Ham. ex Roxburgh) Maxim. (MD)** |  |  |  |
| ***Rostellularia procumbens* (L.) Nees (RP)** |  |  |  |
| ***Polygonum lapathifolium* L. var. salicifolium Sibth. (PL)** |  |  |  |
| ***Lolium perenne* L. (LP)** |  |  |  |
| ***Cichorium intybus* L. (CI)** |  |  |  |
| ***Medicago sativa* L. (MS)** |  |  |  |

The species composition of experimental pots
