# Supplementary material for: Sampling and Complementarity Effects of Plant Diversity on Resource Use Increases the Invasion Resistance of Communities
Source: PLoS One. 2015 Nov 10;10(11):e0141559. doi: 10.1371/journal.pone.0141559 (PMC4640883; doi:10.1371/journal.pone.0141559)
Supplement: S2 Table — (DOC) [file pone.0141559.s002.doc]

**S2 Table.**

| **Names of species** | **Family** |
| --- | --- |
| *Merremia sibirica* (Pers.) Hall. f. | Convolvulaceae |
| *Echinochloa crusgali* (Linn.) Beauv. | Gramineae |
| *Xanthium sibiricum* Patrin ex Widde. | Composite |
| *Plantago asiatica Linn*. | Plantaginaceae |
| *Polygonum persicaria* Linn. | Polygonaceae |
| *Pueraria lobata* (Willd.) Ohwi | Leguminosae |
| *Setaria viridis* (Linn.) Beauv. | Gramineae |
| *Setaria viridis* (Linn.) Beauv. | Gramineae |
| *Bidens pilosa* Linn. | Composite |
| *Solidago canadensis* Linn.* | Composite |
| *Alternanthera philoxeroides* (Mart.) Griseb.* | Amarantaceae |
| *Bidens tripartita* Linn. | Composite |
| *Phragmites australis* (Cav.) Trin. ex Steud. | Gramineae |
| *Rhynchosia volubilis* Lour. | Leguminosae |
| *Humulus scandens* (Lour.) Merr. | Rubiaceae |
| *Kalimeris indica* (Linn.) Sch.-Bip. | Composite |
| *Digitaria sanguinalis* (Linn.) Scop. | Gramineae |
| *Malachium aquaticum*（Linn.）Fries. | Caryophyllaceae |
| *Aster ageratoides* Turcz. Var. scaberulus(Miq.) Ling | Composite |
| *Pterocypsela formosana* (Maxim.) Shih | Composite |
| *Acalypha australis* Linn. | Euphorbiaceae |
| *Conyza canadensis* (Linn.) Cronq.* | Composite |
| *Commelina communis* Linn. | Commelinaceae |
| *Rumex japonicus* Houtt. | Polygonaceae |
| *Crassocephalum crepidioides* (Benth.) S. Moore* | Composite |
| *Erigeron annuus* (Linn.) Pers.* | Composite |
| *Patrinia heterophylla* Bunge | Valerianaceae |
| *Rorippa indica* (Linn.) Hiern | Composite |
| *Aster subulatus* Michx.* | Composite |

The name of non-target native and exotic species

* indicates the exotic species. *Crassocephalum crepidioides* (Benth.) S. Moore is also regarded as an exotic but it is in the China for many years.
